# Factors Affecting the Acceptance of Pandemic Influenza A H1N1 Vaccine amongst Essential Service Providers: A Cross Sectional Study

**DOI:** 10.3390/vaccines1010017

**Published:** 2012-12-20

**Authors:** Alice Beattie, Katie Palmer, Emily Rees, Zoe Riddell, Charlotte Roberts, Rachel Jordan

**Affiliations:** 1 College of Medical & Dental Sciences, University of Birmingham, Edgbaston, Birmingham, B15 2TT, UK; 2 Public Health, Epidemiology & Biostatistics, University of Birmingham, Edgbaston, Birmingham, B15 2TT, UK

**Keywords:** pandemic influenza vaccine, police officers, cross-sectional survey

## Abstract

Although mentioned in the UK pandemic plan, essential service providers were not among the priority groups. They may be important targets of future influenza pandemic vaccination campaigns. Therefore, we conducted a cross-sectional survey among 380 employees from West Midlands police headquarters and 15 operational command units in the West Midlands Area during December 2009–February 2010 to identify factors affecting intention to accept the pandemic influenza A (H1N1) vaccine. One hundred and ninety nine (52.4%) employees completed the questionnaire. 39.7% were willing to accept the vaccine. The most common reasons for intention to accept were worry about catching Swine Flu (n = 42, 53.2%) and about infecting others (n = 40, 50.6%). The most common reason for declination was worry about side effects (n = 45, 57.0%). The most important factor predicting vaccine uptake was previous receipt of seasonal vaccine (OR 7.9 (95% CI 3.4, 18.5)). Employees aged <40 years, males, current smokers, and those who perceived a greater threat and severity of swine flu were also more likely to agree to the vaccine. The findings of this study could be used to improve future pandemic immunization strategies. Targeted education programs should be used to address misconceptions; the single most important factor which might lead to a large improvement in uptake is to allay concern about side effects.

## 1. Introduction

In March 2009 the first cases of pandemic influenza A H1N1 virus were recorded [1]. The World Health Organization classified this outbreak, in June 2009, as phase 6 [2], indicating the start of a global pandemic. As a result of this, countries began implementing their pandemic plans [3]. On 21 October 2009 the UK began its national influenza pandemic vaccination program in preparation for the expected second wave of influenza infections [4].

In comparison to seasonal influenza, the pandemic form was associated with higher hospitalization rates and mortality in younger adults under the age of 65 years, particularly those with underlying medical conditions [5]. It was also associated with more severe disease and increased complications in young children and pregnant women. This was the basis for the selection of target groups vaccinated in the pandemic H1N1 (2009) influenza vaccination program [4]. Although the UK Pandemic Plan stated that one of its goals was to “minimise disruption to health and other essential services” [3], essential service providers (such as the police and fire services) were not included in the priority vaccination groups and were not eventually offered vaccine during the H1N1 pandemic. However, it may be necessary to vaccinate them in future pandemics.

Although uptake of both seasonal and pandemic influenza vaccine is known to be sub-optimal [5,6], the vast majority of studies exploring the determinants of influenza vaccine uptake are among healthcare workers. Doubts about efficacy, inadequate information, perception of not being at risk, vaccine safety and fear of side effects are the most prevalent reasons for vaccine declination [7,8,9,10,11,12].

There is currently no literature addressing the question of vaccination acceptance among essential service providers. In the event of inclusion of this group in a future pandemic influenza vaccination program, it will be critical to understand how to maximize uptake.

The aim of this study, therefore, was to identify the factors contributing to the likely acceptance or declination of pandemic influenza A (H1N1) vaccination among police workers in the West Midlands, UK.

## 2. Experimental Section

### 2.1. Study Design and Setting

A questionnaire-based, cross sectional study among a population of West Midlands (WM) Police employees to identify factors affecting intention to receive pandemic influenza A (H1N1) 2009 vaccine, carried out during the winter pandemic of 2009-10.

### 2.2. Questionnaire Distribution and Sample

Following approval from the Occupational Health Department at the WM Police Headquarters, visits to all 21 Operational Command Units in the West Midlands were made from December 2009 to February 2010. Fifteen centers granted permission to distribute the questionnaires, which were left for one week at each station, before being collected.

Twenty questionnaires were distributed to each participating Operational Command Unit, and 80 questionnaires were distributed in the Occupational Health Department at Police Headquarters to be completed only once by any of the staff employed by the WM Police Service. In total 380 questionnaires were distributed. All questionnaires were self administered and anonymous.

### 2.3. Questionnaire Content

The questions were selected after research of similar literature which provided direction towards potentially significant factors. Standard questions were used where available, these were based upon those used in available literature. A pilot study was carried out on 20 volunteers to improve comprehension.

**Figure A1:**
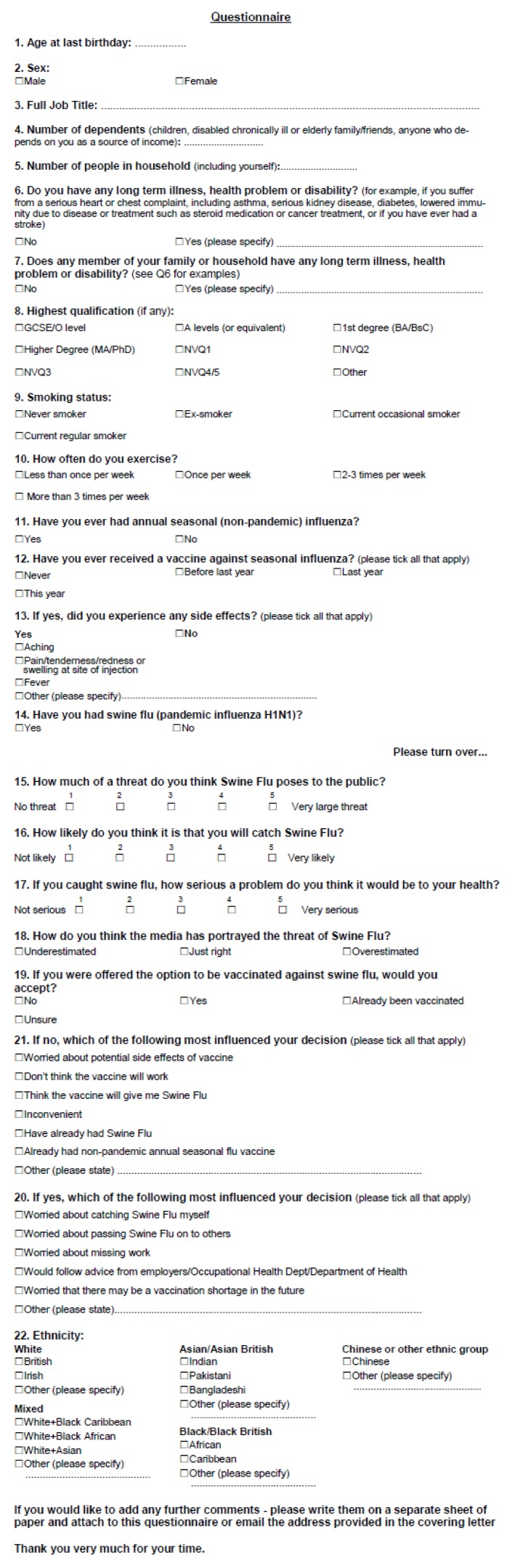
Questionnaire.

The primary outcome of the questionnaire was to determine the intention to have the pandemic influenza A (H1N1) 2009 vaccine. Participants who had already received the vaccine or those who would receive the vaccine if offered were classified as “intending to accept” the vaccine. In addition to this, the questionnaire also collected information on: sociodemographic factors, job title (later classed into office/non-office based jobs), number of dependents, personal or family illness, history of vaccination, history of pandemic influenza A (H1N1) infection, and general health beliefs, in particular those regarding the influenza A (H1N1) pandemic (See Appendix Figure A1).

### 2.4. Statistical Analysis

Data were analyzed using STATA version 11. Univariate associations were analyzed between intention to accept vaccine and important covariates. Factors found to be statistically significant (*p* < 0.005) or clinically important were entered into a multiple logistic regression model. Questions on health beliefs were measured on a 5-point likert scale but collapsed into 3 categories. Scores of 1/2 were classified as low, score 3 as medium, and scores 4/5 as high. Sensitivity analyses were undertaken to exclude those who had received the vaccine already.

## 3. Results

### 3.1. Response Rates

**Figure 1:**
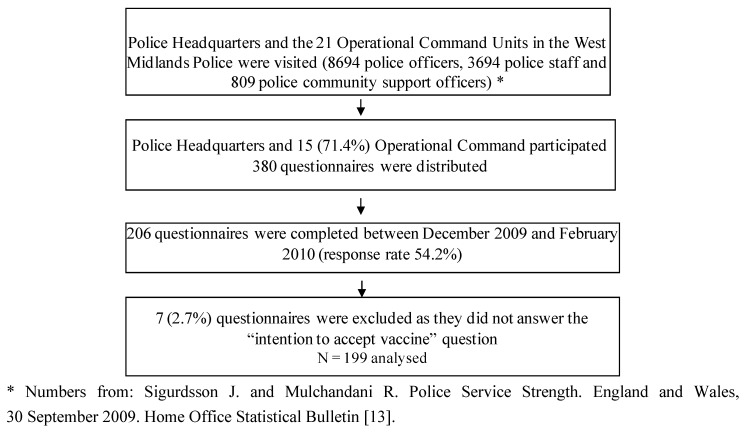
Flow chart illustrating response to questionnaire.

Between December 2009 and February 2010, 380 questionnaires were distributed and 206 completed (response rate 54.2%). Seven questionnaires were excluded as the respondents did not respond to the question about vaccine acceptance, leaving 199 for analysis (Figure 1).

### 3.2. Baseline Characteristics and Descriptive Data

One hundred and five (52.8%) respondents were female (Table 1), their mean age was 38 (Range: 18–63) and 176 (88.4%) were of white ethnicity. The majority had a non-office based job (n = 149, 74.9%) and 57.1% education to at least A’level standard or equivalent. One hundred and twenty four (62.3%) had never smoked although 22 (11.0%) were current smokers. Thirty-two (16.1%) reported a long term illness and more than two-thirds exercised at least once per week. Of the respondents 86 (43.2%) reported seasonal influenza infection in the past, 90 (45.3%) had ever received a seasonal influenza vaccine (39 (43.3% of these reported side effects)). Twenty-eight (14.1%) reported having had “swine flu” during the 2009 pandemic.

* Numbers from: Sigurdsson J. and Mulchandani R. Police Service Strength. England and Wales, 30 September 2009. Home Office Statistical Bulletin [13].

**Table 1:** Characteristics of respondents.

Variable		Total (%)
**Age (years):**		
	<30	44 (22.1)
	30–39	64 (32.2)
	40–49	58 (29.2)
	50–59	23 (11.6)
	>60	7 (3.5)
	*Missing*	*3 (1.5)*
**Sex:**		
	Men	94 (47.2)
	Women	105 (52.8)
**Ethnicity:**		
	White	176 (88.4)
	Mixed	5 (2.5)
	Asian	10 (5.0)
	Black	3 (1.5)
	Chinese	1 (0.5)
	Other	1 (0.5)
	*Missing*	*3 (1.5)*
**Job:**		
	Office Based	46 (23.1)
	Non Office-Based	149 (74.9)
	*Missing*	*4(2.0)*
**Presence of a Long Term Illness in Respondent:**		
	Yes	32 (16.1)
	No	165 (82.9)
	*Missing*	*2 (1.0)*
**Presence of a Long Term Illness in a Family Member of Responder:**		
	Yes	44 (22.1)
	No	153 (76.9)
	*Missing*	*2 (1.0)*
**Highest Qualification:**		
	GCSE/O Level/NVQ1+2	64 (32.1)
	A Level/NVQ3	59 (29.6)
	Degree/NVQ4+5/Higher degree	56 (28.1)
	Other	16 (8.0)
	*Missing*	*4 (2.0)*
**Smoking Status:**		
	Never-Smoker	124 (62.3)
	Ex-Smoker	52 (26.1)
	Current Smoker	22 (11.0)
	*Missing*	*1 (0.5)*
**Exercise Level:**		
	Less than once per week	57 (28.6)
	once a week	33 (16.6)
	2-3 times per week	77 (38.7)
	More than 3 times a week	32 (16.1)
**Ever had Seasonal Flu:**		
	Yes	86 (43.2)
	No	110 (55.3)
	*Missing*	3 (1.5)
**Ever received Seasonal influenza vaccination:**		
	Yes	90 (45.3)
	No	107 (53.8)
	*Missing*	2(1.5)
**Seasonal Vaccine Side Effects:**		
	Yes	39 (43.3)
	No	51 (56.7)
**Have had pandemic influenza A (H1N1) virus :**		
	Yes	28 (14.1)
	No	169 (84.9)
	*Missing*	*2 (1.0)*

Characteristics of the sample were similar to WM police [13] in terms of the age and ethnicity distribution, although in our sample we had many more female respondents than in the police force as a whole. There were fewer smokers in our sample compared with the WM population [14] (data were not available on smoking in the West Midlands police) (Appendix Table A1).

### 3.3. Knowledge and Attitudes of Respondents to Pandemic Influenza A (H1N1) Virus

Eighty-one (40.7%) respondents felt there was a low threat of pandemic influenza A (H1N1) infection to the public, 90 (45.2%) felt there was a medium threat and only 28 (14.1%) felt there was a high threat to the public. Approximately half (n = 94, 49.8%) of respondents believed there was a low probability of catching pandemic influenza A (H1N1) virus and only 12% (n = 24) felt there was a high probability. Correspondingly, most (n = 93, 47.5%) felt the threat to their health was low, while 23.6% (n = 47) felt that the threat to their health was high. The majority of respondents (139, 69.8%) felt the media had overestimated the threat of the pandemic virus (Table 2).

**Table 2:** Respondents’ attitudes to Pandemic Influenza A (H1N1) 2009 virus.

Variable	Total n(%)
**Threat of “Swine Flu” to public:**	
Low	81 (40.7)
Medium	90 (45.2)
High	28 (14.1)
**Likelihood of catching “Swine Flu”:**	
Low	99 (49.7)
Medium	75 (37.7)
High	24 (12.1)
*Missing*	*1 (0.5)*
**Seriousness of “Swine Flu” to Health:**	
Low	93 (46.7)
Medium	56 (28.1)
High	47 (23.6)
*Missing*	*3 (1.5)*
**Media portrayal of threat of “Swine Flu”:**	
Underestimated	4 (2.0)
Just Right	55 (27.6)
Overestimated	139 (69.8)
*Missing*	*1 (0.5)*

### 3.4. Acceptance or Declination of the Pandemic Influenza A (H1N1) Vaccine

Of the 199 respondents, 14 (7.0%) had already received the vaccine, a further 65 (32.7%) said they would accept the vaccine if offered, and 80 (40.2%) said they would decline. 40 (20.1%) were still unsure if they would accept or not. Overall, therefore, intention to receive the vaccine was 79/199 (39.7%). The remaining analyses are based on the 159 respondents who either stated yes or no.

**Figure 2:**
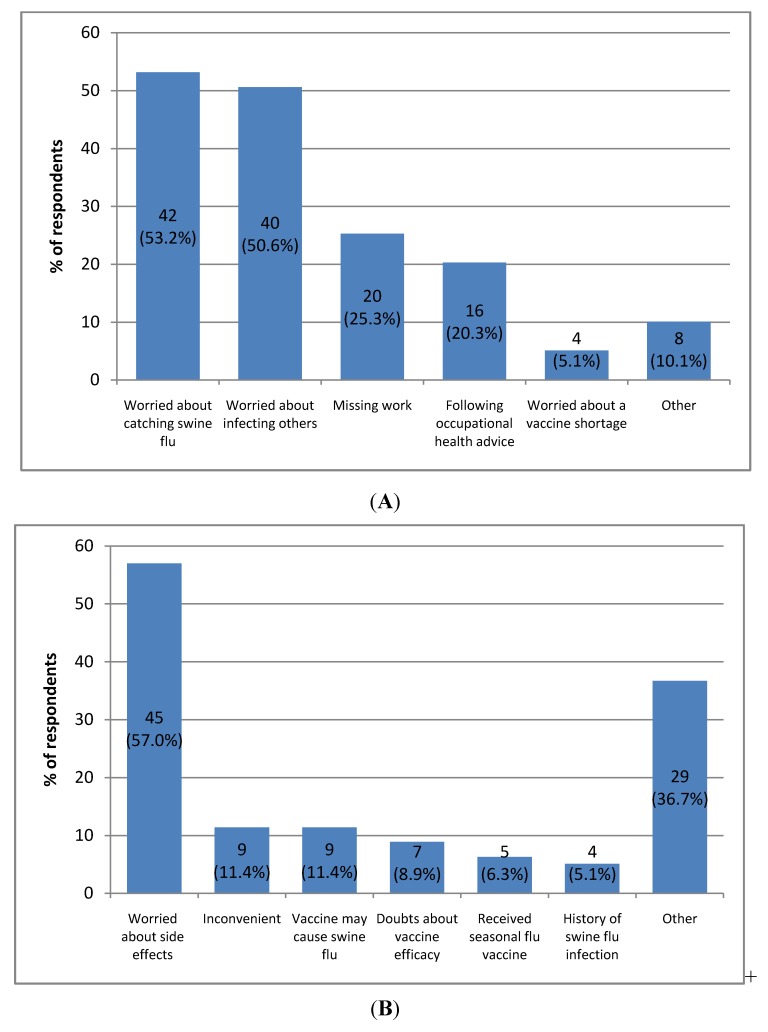
(**A**) Reasons reported by police workers for intention to accept the Pandemic (H1N1) 2009 vaccine; (**B**) Reasons reported by police workers for intention to decline the Pandemic (H1N1) 2009 vaccine.

The most common reasons for accepting the vaccine (Figure 2A) included worry about catching “Swine flu” (n = 42, 53.2%), infecting others (n = 40, 50.6%), and missing work (n = 20, 25.3%) (Figure 2A). Sixteen (20.3%) would accept because they would follow advice from employers/occupational health/Department of Health. The overwhelming reason for declination of the vaccine (Figure 2B) was worry regarding potential side effects (n = 45, 57.0%). However, 9 (11.4%) would/did decline because it was inconvenient, 9 (11.4%) were worried about the vaccine causing “Swine Flu”, 5 (6.3%) would decline because they had already had seasonal influenza vaccine, 7 (8.9%) had doubts about vaccine efficacy and 4 (5.1%) would decline because they had already had pandemic influenza infection that year. Other reasons stated by respondents for declination were: respondents not perceiving themselves to be at risk, simply not wanting to be vaccinated, fate, needle phobia, belief that “Swine flu” is only a threat to those in poor health, or contraindications to the vaccine itself.

### 3.5. Determinants Associated with Intention to Accept the Vaccine

On univariate analysis (Table 3), three determinants related to either demographics or past medical history were significantly associated with the intention to accept the vaccine: non office-based employment (OR: 2.24, 95% CI: 1.06–4.72), having received a seasonal influenza vaccine in the past (OR: 4.08, 95% CI: 2.1–7.93), and history of pandemic influenza A (H1N1) infection (OR: 2.81, 95% CI: 1.03–7.68).

**Table 3:** Determinants associated with intention to accept the Pandemic Influenza A (H1N1) 2009 vaccine.

Variable		Number accepting (%)	OR (95%CI)	Model 1 *	Model 2 *
				Adjusted OR (95% CI)	Adjusted OR (95% CI)
**Age:**					
	<40	42 (52.5%)	1	1	1
	≥40	35 (46.1%)	0.77 (0.41–1.45)	**0.36 (0.16–0.82)**	**0.36 (0.15–0.85)**
**Sex:**					
	Men	43 (56.6%)	1	1	1
	Women	36 (43.4%)	0.59 (0.31–1.10)	**0.47 (0.23–0.99)**	0.53 (0.25–1.12)
**Job:**					
	Office Based	14 (35.1%)	1	-	1
	Non Office-Based	64 (54.7%)	**2.24 (1.06–4.72)**	-	2.05 (0.87–4.86)
**Presence of Long Term Illness:**					
	No	60 (46.9%)	1	-	-
	Yes	17 (58.6%)	1.61 (0.71–3.63)	-	-
**Presence of a Long Term Illness in a Family Member:**					
	No	59 (48.0%)	1	-	-
	Yes	19 (55.9%)	1.37 (0.64–2.95)	-	-
**Highest Qualification:**					
	Below A’level	24 (45.3%)	1	-	-
	A Level equivalent or higher	47 (53.4%)	1.39 (0.70, 2.75)	-	-
**Smoking Status:**					
	Non-Smoker	52 (50.5%)	1	1	1
	Ex-Smoker	17 (40.5%)	0.67 (0.32–1.38)	0.69 (0.30, 1.61)	0.73 (0.31, 1.71)
	Current smoker	10 (71.4%)	2.45(0.72–8.32)	**4.89 (1.05, 22.72)**	4.37 (0.89, 21.35)
**Exercise Level:**					
	Less than once a week	22 (45.8%)	1	-	-
	Once a week	14 (53.9%)	1.37(0.53–3.59)	-	-
	2-3 times a week	30 (51.7%)	1.27(0.59–2.73)	-	-
	More than 3 times a week	13 (48.2%)	1.10(0.43–2.82)	-	-
**Ethnicity:**					
	White	65 (46.8%)	1	-	1
	Non-White	12 (70.6%)	2.66 (0.96–7.31)	-	2.47 (0.73, 8.42)
**Ever received seasonal influenza vaccine**					
	No	27 (33.3%)	1	1	1
	Yes	51 (67.1%)	**4.08 (2.1, 7.93)**	**7.92 (3.38, 18.53)**	**8.59 (3.55, 20.79)**
**Had pandemic influenza A (H1N1) infection:**					
	No	64 (47.1%)	1	-	-
	Yes	15 (71.4%)	**2.81 (1.03–7.68)**	-	-

* Includes 153 observations with complete data; Model 1 adjusted for age, sex, smoking status and prior receipt of influenza vaccine; Model 2 adjusted for age, sex, smoking status, prior receipt of influenza vaccine, ethnicity and type of job; Results in **bold** indicate *p* < 0.05.

Three determinants related to health beliefs and perceptions were significantly associated with the intention to accept the vaccine (Table 4). The belief that “swine flu” posed a high threat to the public was significantly associated with acceptance (OR: 4.64 95% CI: 1.65–13.07). The belief that “swine flu” was a high risk to the respondents’ health was also significantly associated (OR: 3.01 95% CI: 1.36–6.68). In addition, if respondents felt there was a high likelihood of “swine flu” infection, this was significantly associated with intention to accept the vaccine (OR: 4.27 95% CI: 1.42–12.83).

After adjustment (model 1) for age, sex and smoking status, participants who had ever received seasonal influenza vaccine remained significantly more likely to accept the pandemic influenza A (H1N1) vaccine (OR: 7.92, 95% CI 3.38–18.53), with employees over the age of 40 (OR: 0.36 (0.16–0.82), and females (OR: 0.47 (0.23–0.99)) significantly less likely to accept the vaccine, and current smokers more likely (OR: 4.89 (1.05, 22.72)). Additional inclusion of ethnicity and type of job (model 2) suggested a trend towards employees of non-white ethnicity and a job outside the office being more likely to accept the vaccine, although these results were not statistically significant. Excluding participants who had actually received the vaccine (leaving only those stating “intentions”) produced similar results.

Adjustment by age, sex, smoking status and prior receipt of vaccine in a model including determinants related to health beliefs and perceptions highlighted the importance of belief that pandemic influenza A (H1N1) virus was a high threat to the public (OR: 4.44 (1.4, 14.7)), they had a high likelihood of catching the virus (OR: 5.07 (1.44–17.93)) and that “swine flu” was a serious problem to health (OR: 2.86 (1.14–7.15)) to acceptance of the pandemic vaccine (Table 4).

**Table 4:** Determinants related to health beliefs and perceptions associated with intention to accept the Pandemic Influenza A (H1N1) 2009 vaccine.

Variable		Number accepting (%)	OR (95% CI)	Adjusted OR* 95% CI)
**Threat of “Swine Flu” to public:**				
	Low	28 (40.6%)	1	1
	Medium	32 (49.2%)	1.42 (0.72–2.81)	1.86 (0.83, 4.18)
	High	19 (76.0%)	**4.64 (1.65–13.07)**	**4.44 (1.34, 14.70)**
**Likelihood of catching “Swine Flu”:**				
	Low	33 (42.9%)	1	1
	Medium	29 (48.3%)	1.24 (0.63–2.46)	1.38 (0.64–3.00)
	High	16 (76.2%)	**4.27 (1.42–12.83)**	**5.08 (1.44, 17.93)**
**Seriousness of “Swine Flu” to Health:**				
	Low	29 (38.2%)	1	1
	Medium	23 (54.8%)	1.96 (0.91–4.21)	1.43 (0.57–3.56)
	High	26 (65.0%)	**3.01 (1.36–6.68)**	**2.86 (1.14, 7.15)**
**Media portrayal of threat of “Swine Flu”:**				
	Underestimated	3 (75.0%)	1	1
	Just right	29 (65.9%)	0.64 (0.06–6.74)	1.70 (0.12, 24.21)
	Overestimated	47 (42.7%)	0.25 (0.03–2.47)	0.54 (0.04–7.24)

* Includes 153 observations with complete data; Model adjusted for age, sex, smoking status and prior receipt of influenza vaccine; Results in **bold** indicate *p* < 0.05.

## 4. Discussion

### 4.1. Main Findings

This study showed that 39.7% of the police employees we sampled had already been vaccinated or would accept the pandemic Influenza A (H1N1) vaccine. Those who stated that they would decline the vaccine if offered numbered 40.2%, and the remainder were unsure.

#### 4.1.1. Acceptance of Pandemic and Seasonal Influenza Vaccinations

The willingness to receive pandemic Influenza A H1N1 vaccine in this study was found to be 39.7% which is near equivalent to the uptake rate seen in frontline healthcare workers in England of 40.3% in the period leading up to March 2010 [5]. These figures are notably different to those seen in the seasonal influenza vaccination program prior to the pandemic. In healthcare workers, the uptake rate of seasonal vaccine was only 16.5% during the 2008-9 season, compared with over 70% among over 65 year olds [15].

Multivariate analysis identified history of seasonal influenza vaccination as the strongest determinant of positive intention to receive pandemic Influenza A H1N1 vaccine. This result concurs with a number of studies concerning Influenza H5N1 [10,16] and H1N1 [12,17], and also other studies of seasonal influenza uptake [8]. Research into pandemic influenza uptake amongst healthcare workers and general population groups reveals conflicting information about the effects of age on uptake, although generally older employees are more likely to receive vaccine [11,12]. In our study we found that younger employees were more likely to accept. Consistent with the weight of evidence [11], we also found that males were more likely to intend to receive the vaccine. It is possible also that non-white ethnicity (as found in other studies [11]), being a current smoker and working outside of an office environment were positive predictors of vaccine uptake, but small numbers may have limited statistical significance.

#### 4.1.2. Attitudes and Perceptions

Attitudes and perceptions of disease have been shown to determine one’s health protective behaviors, of which immunization is a key example. This is explained by the Health Belief Model [18] (HBM) which states that “*health-related action depends on the simultaneous occurrence of three classes of factors*” which include:

The existence of sufficient health concern or motivation;
Belief of susceptibility to a serious health problem or its complications;
Belief that the benefit of an action outweighs its possible disadvantages.

Our analysis showed that perceived high risk of infection and perceived severity of “Swine Flu” both to one’s health and the public were shown to be significant determinants of acceptance of pandemic Influenza A H1N1 vaccine. This is a prime example of the HBM in clinical practice and concurs with other literature concerning pandemic [11,16,19] and seasonal influenza vaccination [20]. 

The converse of this model is also true, and is demonstrated in our results and those of other authors. [8,10,11,21,22] We found that worry about side effects was four times as influential in determining declination of the vaccine as other factors including: inconvenience, and doubts about safety and efficacy. For these individuals the perceived disadvantages outweigh the benefits, and thus no health protective action, immunization, is taken.

Consistent with most population groups, most respondents rated the pandemic as a medium or low threat, and this is likely to be a major reason for suboptimal vaccine uptake.

### 4.2. Strengths and Limitations of This Study

To our knowledge, this is the only study addressing the issue of vaccine acceptance in essential service providers. The characteristics of our study sample were broadly comparable to the overall WM police [13] (or WM population) [14], although in our sample there were many more women. Since women were less likely to indicate acceptance of the vaccine, we may therefore have underestimated likely uptake rates. Social acceptability and interviewer bias were limited through the use of self administered, anonymous questionnaires.

The small sample size and low response rate limited the statistical power of this study. 380 questionnaires were distributed with a response rate of 54.2% accounting for 2.0% of the sample population. Other than the characteristics stated above there was no way of determining the views of non-responders. Responder bias may have caused an overestimate of vaccine acceptance; those not interested in being vaccinated may have been less likely to participate in the study. This could have been further compounded by lack of promotion of questionnaires.

Questionnaire distribution was targeted at Operational Command Units only; smaller stations were not included in this study. There could be differences between the employees of each. We attempted to counteract this by distributing questionnaires to the Occupational Health Department of WM police where all employees have access. Additionally, use of short questionnaires limited the breadth and depth of responses in certain areas of enquiry.

Our study population was limited to the police force; therefore, our results may not be applicable to those in other essential services.

### 4.3. Implications

The findings of this study can be used to improve immunization strategies if vaccination of essential service providers is implemented in future influenza outbreaks. It is clear that health perceptions and attitudes play a major role in influencing the decision to be vaccinated. Targeted education programs could be implemented to address the misconceptions held by many individuals about health issues and their management; this is particularly applicable in the case of immunization and for pandemic vaccine the key issue appears to be worry about side effects. Wider dissemination of studies depicting accurate portrayal of the side-effect risks and reassurance of the benefits of vaccination could substantially improve uptake among the 40% “decliners” and the 20% “unsures”. Other strategies that have been shown to increase uptake rates among healthcare workers which could be included include: use of mobile services to provide flexible delivery of vaccine [23], opt-out systems [24], and reminder schemes [25].

We also advise that future research is carried out within both the police and fire service, using a larger sample size; this could be achieved through use of employee distribution lists. Active promotion or incentives could be used to increase response rate. Furthermore, it would be beneficial to obtain qualitative data through interviews of study subjects or focus groups.

## 5. Conclusions

Despite some limitations in the power of the study, it is reassuring that acceptance rates of pandemic vaccine among the police force would be at least as good as healthcare workers and substantially better than that seen among healthcare workers in seasonal influenza years. Further exploration of why females and those of older ages (and possibly white ethnicity) are more reluctant to be vaccinated would be useful. In the meantime, it is important to make sure that the public and especially essential workers have confidence in the information about the severity of pandemics, the benefits and disadvantages of vaccination, and that media portrayal is accurate.

## Appendix

**Table A1:** Comparison of Baseline Characteristics between study sample and total WM police [13].

Variable		Study sample (%)	WM Police (%)
Age:			
	≤30	30.6	28.4
	>30	69.4	71.6
Sex:			
	Male	47.1	73.9
	Female	52.9	26.1
Ethnicity:			
	White	89.9	92.7
	Non-white	10.1	7.3
Smoking Status *:			
	Never Smoked	62.9	52.0
	Ex-Smokers	25.9	28.0
	Current Smokers	11.2	20.0

* Data taken from Office of National Statistics due to unavailability of data for West Midlands Police Service [14].
